# 超声波提取-固相萃取-液相色谱-串联质谱法同时测定沉积物中24种皮质类固醇激素

**DOI:** 10.3724/SP.J.1123.2021.03025

**Published:** 2022-02-08

**Authors:** Yongshun ZHOU, Jian GONG, Kexin YANG, Canyuan LIN, Cuiqin WU, Shuhan ZHANG

**Affiliations:** 广州大学环境科学与工程学院, 珠江三角洲水质安全与保护教育部重点实验室, 广东省放射性核素污染控制与资源化重点实验室, 广东 广州 510006; School of Environmental Science and Engineering, Guangzhou University, Key Laboratory for Water Quality and Conservation of the Pearl River Delta, Ministry of Education, Guangdong Provincial Key Laboratory of Radionuclides Pollution Control and Resources, Guangzhou 510006, China; 广州大学环境科学与工程学院, 珠江三角洲水质安全与保护教育部重点实验室, 广东省放射性核素污染控制与资源化重点实验室, 广东 广州 510006; School of Environmental Science and Engineering, Guangzhou University, Key Laboratory for Water Quality and Conservation of the Pearl River Delta, Ministry of Education, Guangdong Provincial Key Laboratory of Radionuclides Pollution Control and Resources, Guangzhou 510006, China; 广州大学环境科学与工程学院, 珠江三角洲水质安全与保护教育部重点实验室, 广东省放射性核素污染控制与资源化重点实验室, 广东 广州 510006; School of Environmental Science and Engineering, Guangzhou University, Key Laboratory for Water Quality and Conservation of the Pearl River Delta, Ministry of Education, Guangdong Provincial Key Laboratory of Radionuclides Pollution Control and Resources, Guangzhou 510006, China; 广州大学环境科学与工程学院, 珠江三角洲水质安全与保护教育部重点实验室, 广东省放射性核素污染控制与资源化重点实验室, 广东 广州 510006; School of Environmental Science and Engineering, Guangzhou University, Key Laboratory for Water Quality and Conservation of the Pearl River Delta, Ministry of Education, Guangdong Provincial Key Laboratory of Radionuclides Pollution Control and Resources, Guangzhou 510006, China; 广州大学环境科学与工程学院, 珠江三角洲水质安全与保护教育部重点实验室, 广东省放射性核素污染控制与资源化重点实验室, 广东 广州 510006; School of Environmental Science and Engineering, Guangzhou University, Key Laboratory for Water Quality and Conservation of the Pearl River Delta, Ministry of Education, Guangdong Provincial Key Laboratory of Radionuclides Pollution Control and Resources, Guangzhou 510006, China; 广州大学环境科学与工程学院, 珠江三角洲水质安全与保护教育部重点实验室, 广东省放射性核素污染控制与资源化重点实验室, 广东 广州 510006; School of Environmental Science and Engineering, Guangzhou University, Key Laboratory for Water Quality and Conservation of the Pearl River Delta, Ministry of Education, Guangdong Provincial Key Laboratory of Radionuclides Pollution Control and Resources, Guangzhou 510006, China

**Keywords:** 超声波提取, 固相萃取, 超高效液相色谱-串联质谱, 皮质类固醇激素, 沉积物, ultrasonic extraction, solid phase extraction (SPE), ultra performance liquid chromatography-tandem mass spectrometry (UPLC-MS/MS), corticosteroids, sediments

## Abstract

沉积物中皮质类固醇激素(CSs)的痕量分析对探究其在环境多介质中的赋存状况和环境行为具有重要意义。然而目前相关研究主要集中在水样中糖皮质激素的测定,对基质更为复杂的环境固体样品中CSs的定量分析研究仍十分有限亦缺乏针对性、系统性,且目标物未能覆盖大多数常见常用的糖/盐CSs。本研究系统地优化了样品前处理过程和仪器分析中影响24种CSs测定准确性和灵敏度的条件,建立了采用超声波提取联合固相萃取技术对样品进行前处理,利用超高效液相色谱-串联质谱(UPLC-MS/MS)同时测定沉积物中24种CSs的分析方法。沉积物经冷冻干燥、研磨后,取2.0 g样品采用甲醇-丙酮(1:1, v/v)超声提取,HLB固相萃取柱富集、净化,LC-NH_2_小柱二次净化,甲醇定容。目标物经Agilent ZORBAX Eclipse Plus C_8_反相色谱柱(100 mm×2.1 mm, 1.8 μm)分离,柱温30 ℃,进样量5 μL,以乙腈和0.1%(v/v)乙酸水溶液作为流动相进行梯度洗脱,流速0.3 mL/min。在电喷雾正离子模式(ESI^+^)下采用动态多反应(DMRM)选择离子监测(SIM)方式测定24种目标化合物,内标法定量。结果表明,24种CSs的方法检出限(LOD, *S/N*≥3)和定量限(LOQ, *S/N*≥10)分别为0.14~1.25 μg/kg和0.26~2.26 μg/kg,工作曲线在1.0~100 μg/L范围内具有良好的线性关系(*R*^2^>0.995)。在5、20、50 μg/kg的基质加标水平下,24种CSs的平均回收率为64.9%~125.1%,相对标准偏差为0.4%~12.6%(*n*=5)。应用该方法测定了3份珠江三角洲河流沉积物,其中有11种目标物被检出,含量范围为1.25~29.38 μg/kg。该方法净化效率高,灵敏可靠,适用于环境沉积物中多种天然和合成CSs的痕量检测。

## Abstract (en)

Corticosteroids (CSs) are widely used to treat various inflammatory and immune diseases in humans and animals, such as arthritis and lupus. Thus far, CSs have been frequently detected in diverse pollution sources, such as in the influent and effluent of traditional wastewater treatment plants, livestock farms, and aquaculture. Owing to incomplete removal or limited treatment, CSs can enter the water environment and eventually be adsorbed in the sediment. Due to hydrodynamic effects, CSs can re-enter the surface water through the resuspension of sediments, and pose a hazard to the ecosystem and human health via the enrichment of aquatic organisms and transmission through the food chain. Therefore, trace analysis of CSs in sediments is significant for exploring their prevalence and behavior in multiple environments. However, existing research mainly focuses on the determination of glucocorticoids in water samples, and studies on the systematic quantitative analysis of CSs in environmental solid samples with more complex matrices are scarce. Moreover, majority of previous investigations focused on a limited number of glucocorticoids, making it important to widen the range of target compounds to be studied, including mineralocorticoids.

In this study, the main factors which could influence the accuracy and sensitivity in the determination of 24 target CSs were systematically optimized in the sample pretreatment and instrument analysis. A novel method based on ultrasonic extraction coupled with solid phase extraction (SPE) for sample pretreatment was developed for the simultaneous determination of the 24 CSs in sediments using ultra-performance liquid chromatography-tandem mass spectrometry (UPLC-MS/MS). The sediment sample was ground to homogenize the particle sizes after freeze-drying. The analytes from 2.0 g of the sample were ultrasonicated and extracted with methanol-acetone (1:1, v/v). After concentrating and diluting each extract, SPE was performed. The water sample was extracted and purified using hydrophile-lipophile balance (HLB) cartridges, following which the extract was further purified with LC-NH_2_ cartridges. The extracts were concentrated using a rotary evaporator, dried under a gentle stream of nitrogen, and re-dissolved in methanol for instrumental analysis. Chromatographic separation was conducted on an Agilent ZORBAX Eclipse Plus C_8_ column (100 mm×2.1 mm, 1.8 μm), with a column flow rate of 0.3 mL/min and a gradient of mobile phases A (water with 0.1% acetic acid) and B (acetonitrile). The column temperature was set to 30 ℃ and the injection volume was fixed at 5 μL. Electrospray ionization MS in the dynamic multiple reaction monitoring (DMRM) and selected ion monitoring (SIM) modes were performed in the positive mode for the qualitative and quantitative analysis of the target compounds. Quantitation of the target compounds was carried out using the internal standard method. The effects of different extraction solvents, purification conditions, and MS conditions on the recoveries of the target compounds were investigated. The limits of detection (LODs) (*S/N*≥3) and limits of quantification (LOQs) (*S/N*≥10) of all 24 compounds were in the ranges of 0.14-1.25 μg/kg and 0.26-2.26 μg/kg, respectively. The correlation coefficients of linear calibration curves were higher than 0.995 in the range of 1.0-100 μg/L. The recoveries of the 24 CSs at 5, 20, and 50 μg/kg spiked levels ranged from 64.9% to 125.1% with relative standard deviations of 0.4%-12.6% (*n*=5). The developed method was applied to analyze the CSs in three sediment samples from the rivers of the Pearl River Delta. In all, 11 target compounds were detected in these samples, with contents in the range of 1.25-29.38 μg/kg. The characteristic of this method is efficient, sensitive, reliable, and suitable for the trace determination of varieties of natural and synthesized CSs in environmental sediments.

皮质类固醇激素(corticosteroids, CSs)是一类由肾上腺皮质所合成的重要激素,几乎参与了人类和动物的所有生命活动^[1,2]^。人工合成的CSs类药物被广泛应用于炎症、免疫类疾病治疗^[3,4]^,但长期使用可能导致人体免疫力下降、肥胖等疾病^[5]^。CSs可通过人体和动物的排泄、污水处理厂的不完全处理排放,并最终进入天然水环境中。相关研究表明多种CSs普遍存在于环境水体中,可能已对水生系统造成不利影响^[6,7]^。与其他有机污染物一样,CSs可通过吸附作用附着于悬浮颗粒上并随之迁移,最终沉积到水底,富集于沉积物中;也可在水动力或其他理化条件变化时重新释放到水体,并通过生物富集和食物链的传递作用进而危害生态系统和人类健康^[8]^。迄今为止,关于环境中CSs污染现状的研究主要集中在地表水和污水处理厂上^[9,10]^,而对于其在沉积物等天然固相介质中的赋存状况则鲜有报道^[11]^。

环境中CSs的残留水平不高(μg/L至ng/L级),沉积物样品基质组成复杂,前处理难度较大,因此,目前关于沉积物中CSs的残留分析方法仍十分有限。有关CSs在天然环境中的含量水平、污染特征等研究刚起步^[9,10,11]^,对该类污染物环境地球化学行为的认识亦非常有限。这主要受制于缺少相关的分析方法,特别是缺少针对基质复杂的环境介质中CSs多残留的痕量分析方法。Zhang等^[12]^测定了基质较为复杂的市政污水中3种糖皮质激素,然而该方法回收率较低,检出限和定量限较高,且实际样品中未能检测出目标物;Liu等^[13]^和Chen等^[14]^分析了水体颗粒物和沉积物中4~5种糖皮质激素,仅有皮质醇被检出。虽然之前Liu等^[15]^、杨雷等^[16]^、Fan等^[17]^、Weizel等^[18]^开展了沉积物和污泥中数十种类固醇激素的监测,但就CSs而言该类分析方法缺乏针对性和系统性。而且其目标物仅包含5~12种糖皮质激素,不仅未涉及盐皮质激素,也未能覆盖大多常见常用的CSs。水环境中沉积物既是污染物的“汇”也是“源”,因此,有必要针对性地发展一套简便、灵敏的沉积物中CSs多残留的痕量分析方法,并将其应用到实际的环境监测中,为更系统地研究CSs在环境多介质中的赋存状况和环境行为提供技术保障。

针对基质更为复杂的环境沉积物,本研究在水样分析方法^[19]^的基础上,进一步采用超声波辅助提取联合固相萃取(SPE)技术对样品进行前处理,并对提取液开展二次深度净化降低基质干扰;重新对质谱条件进行优化,兼顾信号响应和仪器耐受性。最终建立了一套利用超高效液相色谱-串联质谱(UPLC-MS/MS)同时检测沉积物中24种CSs的定量分析方法,并应用于珠江沉积物的CSs测定。

## 1 实验部分

### 1.1 仪器、试剂与材料

1260 Infinity-6460 QQQ超高效液相色谱-三重四极杆质谱联用系统(UPLC-MS/MS, Agilent公司)及Agilent MassHunter定量分析软件;Autotrace280全自动固相萃取仪(美国Thermo公司); Rotavapor R-120旋转蒸发仪(瑞士BUCHI公司); EFAA-DC12氮吹仪(上海安谱公司); Oasis HLB SPE柱(500 mg, 6 mL, Waters公司); Silica SPE柱(500 mg, 6 mL, Waters公司); LC-NH_2_ SPE柱(500 mg, 3 mL, Supelco公司)。

24种CSs标准样品(见表1)均购自美国Sigma-Aldrich公司,纯度均大于98%; 5种同位素替代物标准品:氢化可的松-d4、地塞米松-d4、曲安奈德-^13^C3、布地奈德-d8、丙酸氟替卡松-d5均购自加拿大Toronto Research Chemicals公司,纯度均大于95%;甲醇、乙酸乙酯、乙腈、丙酮(HPLC级,德国CNW公司);实验用水均为超纯水(电阻率18.25 MΩ·cm)。

**表 1:** 24种目标化合物的质谱参数

No.	Compound	Retention time/min	Monitoring ion pair (m/z)	Fragmentor/V	Collision energy/eV
1	triamcinolone	2.61	395.2/375.1^*^	140	4
	(曲安西龙)		395.2/225.1		12
2	aldosterone	4.51	361.0/315.0	70	8
	(醛固酮)		361.0/343.0^*^		4
3	prednisolone	5.73	361.2/343.2^*^	70	4
	(泼尼松龙)		361.2/147.1		24
4	cortisol	6.11	363.2/327.2	95	12
	(皮质醇)		363.2/121.0^*^	120	27
5	prednisone	6.19	359.2/171.0	95	36
	(泼尼松)		359.2/147.0^*^		32
6	cortisone	6.76	361.2/163.1^*^	150	12
	(可的松)		361.2/121.0		4
7	methylprednisolone	10.23	375.2/357.1^*^	110	6
	(甲基泼尼松龙)		375.2/161.1		20
8	betamethasone	11.13	393.2/373.2^*^	75	0
	(倍他米松)		393.2/355.2		8
9	dexamethasone	11.25	393.2/373.2^*^	75	0
	(地塞米松)		393.2/355.2		8
10	flumethasone	11.59	411.2/253.1^*^	70	12
	(氟米松)		411.2/121.0		40
11	corticosterone	11.63	347.0/121.0^*^	20	25
	(皮质酮)		347.0/329.0		25
12	beclomethasone	11.86	409.2/391.1^*^	110	6
	(倍氯米松)		409.2/146.9		30
13	flunisolide	12.40	495.2/121.0^*^	90	16
	(氟尼缩松)		495.2/319.1		44
14	triamcinolone acetonide	12.52	435.2/415.2^*^	75	4
	(曲安奈德)		435.2/397.2		12
15	fluocinolone acetonide	13.34	453.2/413.3^*^	90	8
	(醋酸氟轻松)		453.2/337.2		8
16	fluorometholone	13.71	377.2/121.0^*^	80	36
	(氟米龙)		377.2/173.1		24
17	fludrocortisone acetate	13.84	423.2/238.9^*^	150	22
	(醋酸氟氢可的松)		432.2/120.9		36
18	deflazacort	14.04	442.2/123.9^*^	170	50
	(地夫可特)		442.2/141.9		36
19	budesonide	15.43	431.2/147.0^*^	90	36
	(布地奈德)		431.1/173.1		28
20	deoxycorticosterone acetate	17.20	373.24/97.1^*^	110	28
	(醋酸脱氧皮质酮)		373.2/108.9		20
21	amcinonide	17.36	503.2/339.1^*^	75	12
	(安西奈德)		503.2/399.2		8
22	clobetasol propionate	17.61	467.2/373.1	65	8
	(丙酸氯倍他索)		467.2/355.1^*^		8
23	fluticasone propionate	17.79	501.2/312.9^*^	110	24
	(丙酸氟替卡松)		501.2/292.9		44
24	clobetasone butyrate	18.84	479.2/279.1	80	16
	(丁酸氯倍他松)		479.2/71.1^*^		16
	cortisol-d4	6.15	367.2/121.0^*^	120	27
	dexamethasone-d4	11.36	397.2/377.2^*^	75	0
	triamcinoloneacetonide-^13^C3	12.39	438.2/318.2^*^	75	4
	budesonide-d8	15.33	439.0/421.0^*^	110	12
	fluticasone propionate-d5	17.83	506.2/312.9^*^	110	24

* Quantitation ion.

实际样品为珠江三角洲河流表层沉积物。

### 1.2 标准溶液的配制

首先称取适量标准品,用甲醇配制成质量浓度为100 mg/L的单标储备液。取适量储备液用甲醇稀释配制成10 mg/L的24种目标物的混合标准溶液和10 mg/L的5种同位素替代物混合标准溶液。然后用甲醇逐级稀释至质量浓度为1.0~100 μg/L的系列混合标准工作溶液;另取适量的5种同位素替代物混合标准溶液稀释成1 mg/L的内标工作液备用。所有标准溶液置于冰箱-20 ℃避光储存。

### 1.3 样品的采集与处理

用不锈钢抓斗式采样器从珠江三角洲河流采集表层(0~20 cm)沉积物样品,转移至实验室并置于-20 ℃冷冻保存至分析。

称取2.0 g经冷冻干燥、研磨过筛的沉积物样品置于50 mL具塞聚丙烯离心管中,加入0.5 g铜片、10 mL甲醇-丙酮(1∶1, v/v)、20 μL的内标工作液,混匀后超声提取10 min,再以6000 r/min速度离心10 min并收集上层清液。上述步骤重复3次并合并提取液。利用旋转蒸发仪将提取液浓缩至1 mL后,溶于200 mL超纯水中,溶液用于固相萃取。Oasis HLB小柱依次用6 mL乙酸乙酯、6 mL乙腈、12 mL超纯水活化后,使用全自动固相萃取仪以10 mL/min的流速将试样溶液过柱。上样完毕后,先用10 mL 10%(v/v)乙腈水溶液淋洗,接着氮吹干燥柱床,然后依次用6 mL乙酸乙酯-乙腈(1∶1, v/v)、6 mL乙酸乙酯洗脱。收集的洗脱液经旋转蒸发、氮气吹干后复溶于1 mL甲醇。用5 mL乙酸乙酯、5 mL甲醇活化LC-NH_2_小柱,再将甲醇浓缩液过柱净化,用5 mL甲醇洗脱。用氮气将洗脱液吹至近干并以甲醇定容至0.5 mL,待UPLC-MS/MS测定。

### 1.4 色谱条件

Agilent ZORBAX Eclipse Plus C_8_色谱柱(100 mm×2.1 mm, 1.8 μm);流动相A: 0.1%(v/v)乙酸水溶液,流动相B:乙腈;柱温30 ℃;流速0.3 mL/min;进样量5 μL;梯度洗脱程序^[19]^: 0~8.0 min, 28%B; 8.0~8.1 min, 28%B ~ 40%B; 8.1~12.0 min, 40%B; 12.0~12.1 min, 40%B~60%B; 12.1~16.0 min, 60%B~70%B; 16.0~16.5 min, 70%B~100%B; 16.5~20.5 min, 100%B。

### 1.5 质谱条件

离子源:电喷雾电离源,正离子扫描模式(ESI^+^);雾化器压力:276 kPa;脱溶剂气流速:11 L/min;离子源温度:300 ℃;毛细管电压4000 V;采集时间窗口(delta retention time)为1.2 min,采用动态多反应选择性监测扫描模式(DMRM)。优化后的24种目标化合物的质谱参数见表1。

定量分析:为了有效监控实验过程的系统误差,消减基质效应的影响,提高定量结果的准确性,本研究采用内标法定量。使用5种同位素替代物作为内标对24种目标物进行定量分析:皮质醇-d4(No.1~6)、地塞米松-d4(No.7~12)、曲安奈德-^13^C3(No.13~18)、布地奈德-d8(No.19~20)、丙酸氟替卡松-d5(No.21~24)(括号中为其对应的表1中目标物的编号)。

## 2 结果与讨论

### 2.1 提取溶剂的选择

选择合适的萃取溶剂能够提高目标物的提取效率同时减少杂质干扰。考虑到目标化合物种类较多、极性范围较大且沉积物样品基质复杂等特点,本研究选取弱、中、强极性的3种常用溶剂乙酸乙酯、乙酸乙酯-乙腈(1∶1, v/v)以及甲醇-丙酮(1∶1, v/v),用于样品超声萃取并比较其提取效率。

3种溶剂萃取加标样品的回收率结果见图1。可以看出,样品经乙酸乙酯溶剂提取后,所有目标物均被检出,回收率为8.58%~154.4%,相对标准偏差(RSD)为1.28%~40.8%;乙酸乙酯-乙腈(1∶1, v/v)的提取液中未能检测出曲安西龙,其余目标物回收率为6.19%~172.2%,RSD为0.64%~21.3%;样品经甲醇-丙酮(1∶1, v/v)提取后所有目标物均被检出,回收率为71.7%~130.2%,RSD为1.1%~9.7%。与前两者相比,甲醇-丙酮(1∶1, v/v)作为提取溶剂具有较高的回收率和重复性。因此本研究选择甲醇-丙酮(1∶1, v/v)作为超声萃取溶剂。

**图1:**
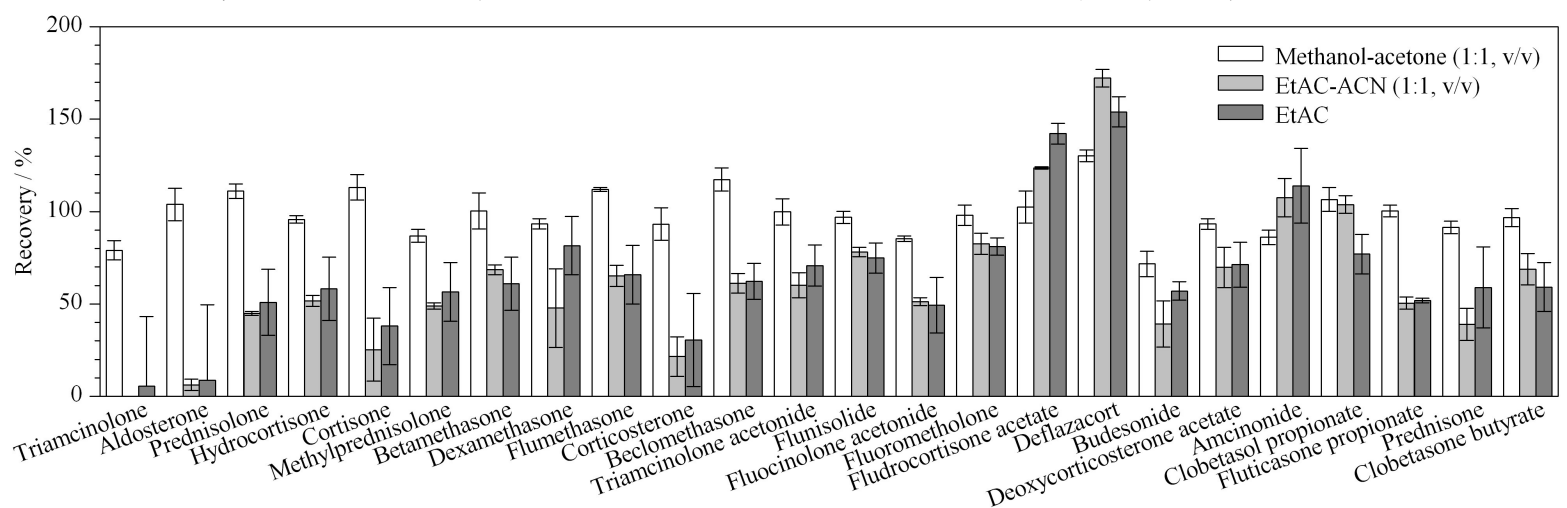
提取试剂对24种皮质类固醇激素回收率的影响(*n*=5)

### 2.2 SPE净化条件的选择及优化

沉积物样品基质复杂,其提取液中难免含有大量的干扰物,不但影响仪器分析的准确性,还会缩短分离柱的使用寿命^[20]^。因此为尽量降低基质效应的影响,本研究考察了Silica柱和LC-NH_2_柱对SPE浓缩液作进一步净化的效果。其中Silica柱是以硅胶为基质的极性吸附剂,主要用于分离非水溶液中的低极性组分^[21]^,通过增加极性高的洗脱溶剂将结构相似的化合物分开;LC-NH_2_柱是以氨丙基为基质的中等极性吸附剂,能够从极性溶液中萃取非极性化合物,与Silica柱一样适用于分离异构体^[22]^。

基于先前的研究^[9]^,采用Oasis HLB SPE柱对样品提取液进行富集并用10 mL的10%乙腈水溶液洗柱,作为第一次净化。接着再分别用上述两种净化柱对HLB柱洗脱液进行第二次净化:(1)洗脱液浓缩后溶于1 mL乙酸乙酯-正己烷(1∶9, v/v),并转移至活化的Silica柱,并用3 mL乙酸乙酯-正己烷(1∶9, v/v)淋洗,最后用4 mL乙酸乙酯-甲醇(95∶5, v/v)洗脱,洗脱液经旋转蒸发、温和氮气吹干后用甲醇定容,待测;(2)洗脱液浓缩后溶于1 mL甲醇,并转移至活化的LC-NH_2_柱,用5 mL甲醇洗脱LC-NH_2_柱,洗脱液经旋转蒸发、温和氮气吹干,甲醇定容后待测。通过比较只经过HLB柱一次净化以及分别再经Silica柱、LC-NH_2_柱二次净化的3种情况,对净化效果进行综合评估。

3种净化条件下目标物回收率结果如图2所示。样品仅经过HLB柱一次净化时,目标化合物的回收率普遍偏低(25.5%~95.9%)且RSD范围较大(11.1%~42.0%),重现性较差。浓缩液经Silica柱二次净化后,目标物之一曲安西龙并未检出,可能与其在硅胶柱中保留较强,不易被洗脱下来有关。其余目标化合物的回收率为52.7%~94.7%, RSD为0.9%~8.6%。浓缩液经LC-NH_2_柱二次净化后,所有目标化合物均被检出,回收率为61.4%~118.4%, RSD为0.8%~6.5%。可见其净化效果优于前两种情况且回收率高、重现性较好。对比浓缩液经过LC-NH_2_柱净化前后代表性化合物的信号响应(见图3)发现,净化后的样品中基质干扰明显降低,目标物信号增强。因此本研究选用LC-NH_2_柱对样品进行二次净化。

**图2:**
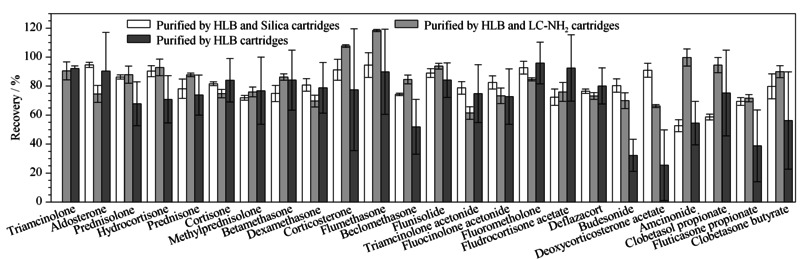
SPE净化条件对24种皮质类固醇激素回收率的影响(*n*=5)

**图3:**
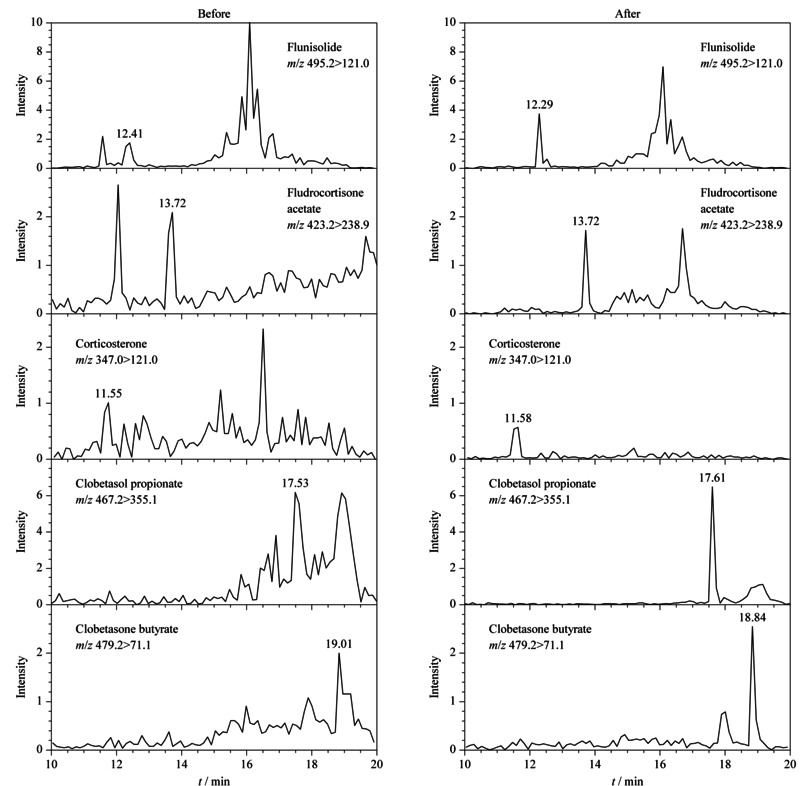
氟尼缩松、醋酸氟氢可的松、皮质酮、丙酸氯倍他索和丁酸倍他松经LC-NH_2_ SPE柱净化前、后的MRM色谱图

### 2.3 质谱条件的优化

CSs是典型的甾体类化合物,其基本结构如图4所示。化合物在C-17、C-20和C-21处存在酮基或羟基,能够在ESI^-^模式下离子化,产生的母离子为[M+HCOO]^-^;在C-3处存在共轭羰基等多电子基团,所以在ESI^+^模式下能形成[M+H]^+^母离子。因此CSs可以在正、负两种电离模式下检测到。通过单标溶液进样,分别对24种目标物的质谱参数进行优化。首先,通过母离子的全扫描(full scan)确定化合物的准分子离子,然后在选择离子监测(SIM)模式下优化碎裂电压和毛细管电压,使[M+H]^+^或[M+HCOO]^-^响应达到最大;然后对母离子作子离子全扫描(product ion scan),选择2个具有更高丰度的特征碎片离子,优化碰撞能量以使响应最大化,最后获得响应最佳的两对MRM离子对和相应的质谱参数。

**图4:**
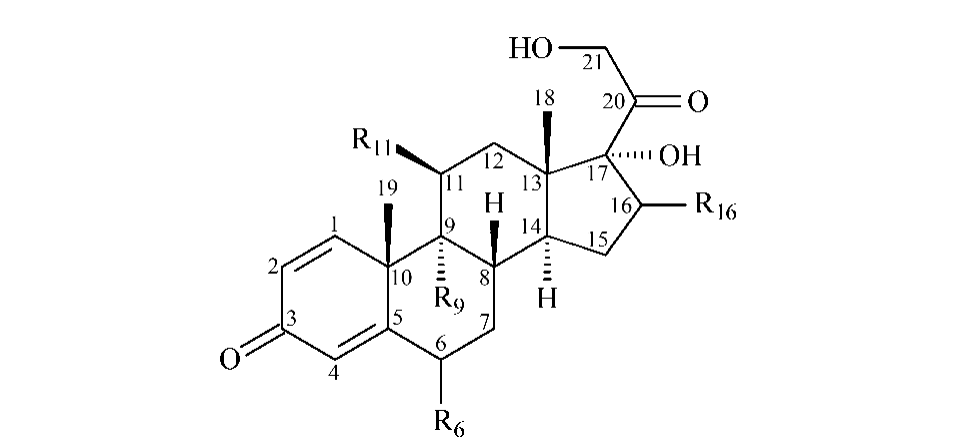
皮质类固醇激素的结构骨架

分别在ESI^-^、ESI^+^和ESI^±^模式下,对24种目标物的特征离子进行扫描。结果发现,在ESI^-^模式下,皮质醇、泼尼松、皮质酮等十几种目标物的信号强度过低^[23]^;在ESI^+^模式下的大部分化合物基峰的相对丰度比ESI^±^模式高出2~6倍(见图5)。另外,考虑到ESI^±^模式下须使用导电毛细管,与ESI^+^模式下使用的石英毛细管相比,其耐脏程度低、使用寿命也较短。因此,本研究选择石英毛细管并在ESI^+^下对目标化合物进行分析。

**图5:**
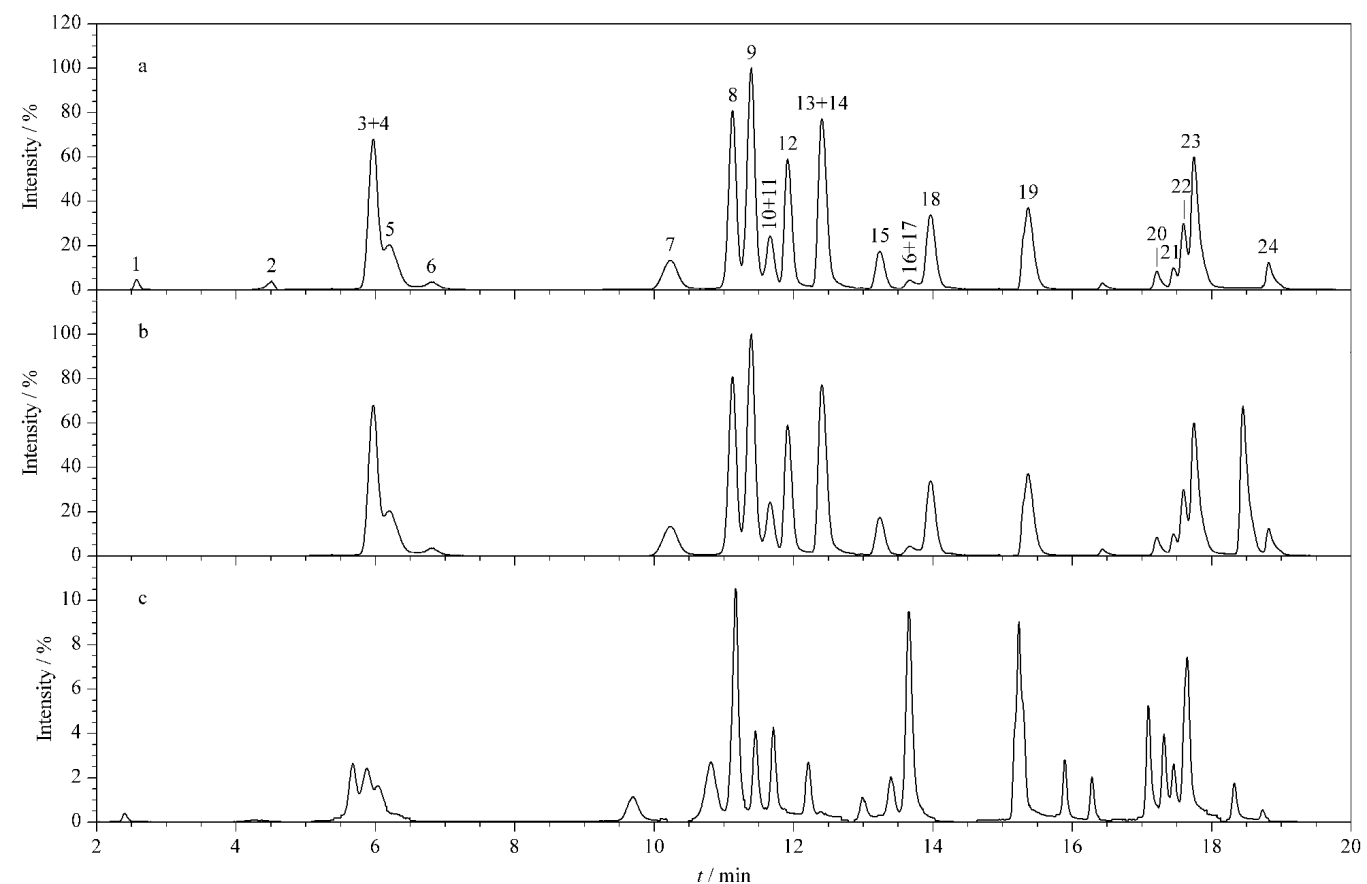
24种目标物混合标准溶液(20 μg/L)在(a)正离子模式、(b)负离子模式、和(c)正负离子模式下的总离子色谱图

### 2.4 基质效应评价

沉积物样品基质复杂,进行质谱分析时易受到基质干扰,影响方法的灵敏度和重现性。本研究采用向沉积物提取液和纯溶剂中加标的方式评价基质效应(ME)的影响,计算公式如下^[24]^:ME=(1-扣除本底后基质溶液中目标物的响应值/纯溶剂中相应目标物的响应值)×100%。

结果(见表2)发现,沉积物样品在未经LC-NH_2_柱净化的条件下,目标物受基质干扰的影响大,ME值范围在-7.7%~74.5%之间,大部分化合物表现为较强的基质抑制作用;利用LC-NH_2_柱对沉积物样品进行净化后,ME值范围为-7.9%~27.9%,大多数目标物基质效应在±20%以内,表明该净化步骤可有效降低基质干扰。为进一步消减基质效应的影响,本研究采用同位素内标法定量。这不但能有效监控分析过程,消除系统误差,也保证了定量结果的准确度。

**表 2:** 24种目标化合物的线性方程、相关系数、检出限、定量限和基质效应

Compound	Linear equation	*R* ^2^	LOD/ (μg/kg)	LOQ/ (μg/kg)	MEs/%	
					a	b
Triamcinolone	*y*=1.10×10^3^*x*+6.44×10^4^	0.999	1.06	2	-7.7	5.7
Aldosterone	*y*=3.52×10^3^*x*-3.41×10^3^	0.999	0.50	2.26	20.3	16.5
Prednisolone	*y*=1.10×10^4^*x*-8.32×10^4^	0.999	1.25	1.82	32.9	11.6
Cortisol	*y*=1.29×10^3^*x*-7.79×10^2^	0.999	0.27	1.14	31.5	23.4
Prednisone	*y*=7.64×10^2^*x*+3.63×10^2^	0.998	0.33	0.91	26.2	18.8
Cortisone	*y*=6.78×10^2^*x*-3.82×10^2^	0.999	0.17	0.26	16.0	10.7
Methylprednisolone	*y*=3.42×10^3^*x*-1.37×10^3^	0.999	0.45	1.20	26.3	16.3
Betamethasone	*y*=2.55×10^4^*x*-1.54×10^4^	0.999	0.47	0.93	15.8	10.4
Dexamethasone	*y*=2.76×10^4^*x*-2.14×10^4^	0.999	0.50	1.35	21.3	14.7
Flumethasone	*y*=1.31×10^3^*x*+8.83×10^2^	0.999	0.47	1.14	10.1	-4.3
Corticosterone	*y*=2.17×10^3^*x*-2.05×10^3^	0.999	0.48	1.18	22.7	14.3
Beclomethasone	*y*=1.31×10^4^*x*-7.56×10^2^	0.998	0.57	1.45	49.9	18.2
Flunisolide	*y*=1.45×10^2^*x*+3.48×10^3^	0.999	0.57	1.55	19.3	-7.9
Triamcinolone acetonide	*y*=3.55×10^3^*x*+8.79×10^2^	0.998	0.63	1.63	34.8	21.8
Fluocinolone acetonide	*y*=2.42×10^3^*x*-1.46×10^3^	0.999	0.29	0.88	28.0	-4.4
Fluorometholone	*y*=5.89×10^3^*x*-1.61×10^4^	0.995	0.60	1.69	21.2	11.4
Fludrocortisone acetate	*y*=5.62×10^2^*x*+6.41×10^2^	0.997	0.58	1.37	19.6	15.9
Deflazacort	*y*=1.45×10^4^*x*-1.02×10^4^	0.997	0.14	0.33	31.1	25.2
Budesonide	*y*=2.16×10^3^*x*+3.61×10^2^	0.997	0.51	1.31	71.8	24.3
Deoxycorticosterone acetate	*y*=8.20×10^3^*x*+5.04×10^2^	0.999	0.42	0.98	74.5	27.5
Amcinonide	*y*=6.01×10^3^*x*-2.38×10^3^	0.998	0.57	1.16	45.5	-3.4
Clobetasol propionate	*y*=3.55×10^3^*x*-3.09×10^3^	0.998	0.42	1.64	24.7	-0.9
Fluticasone propionate	*y*=1.12×10^3^*x*+5.60×10^2^	0.995	0.47	1.44	61.3	27.9
Clobetasone butyrate	*y*=3.64×10^3^*x*-3.17×10^3^	0.999	0.56	1.48	43.7	9.3

*y*: peak area ratio of analyte to internal standard; *x*: mass concentration of analyte, μg/L. a: ME values without the secondary purification by LC-NH_2_ SPE column; b: ME values with secondary purification by LC-NH_2_ SPE column.

### 2.5 标准曲线的线性方程、检出限和定量限

配制1.0~100 μg/L的系列混合标准溶液,同时加入5种同位素内标,质量浓度均为10 μg/L。按照浓度由低到高依次进样,以待测物与内标物定量离子的峰面积比值为纵坐标,待测物的质量浓度(μg/L)为横坐标,分别绘制标准曲线。24种CSs具有良好的线性关系,相关系数(*r*^2^)均大于0.995。按信噪比(*S/N*)=3和*S/N*=10计算方法的检出限(LOD)和定量限(LOQ),所有化合物的LOD和LOQ分别在0.14~1.25 μg/kg和0.26~2.26 μg/kg范围内(见表2)。

### 2.6 加标回收率和精密度

依据目前有限的报道^[11]^,沉积物中CSs的含量约为类固醇雌激素的几倍至数十倍,而珠江流域沉积物中的类固醇雌激素残留普遍在几个μg/kg水平^[25]^。因此,本文分别设定了5、20、50 μg/kg 3个加标水平,取2 g沉积物并添加不同量的混合标准溶液,每个加标水平重复测定5次。结果如表3所示,样品平均加标回收率(*n*=5)为64.9%~125.1%, RSD(*n*=5)为0.4%~12.6%,均满足环境样品有机物痕量检测要求。

**表 3:** 沉积物中24种皮质类固醇激素的加标回收率及精密度(*n*=5)

Compound	5 μg/kg			20 μg/kg			50 μg/kg	
	Recovery/%	RSD/%		Recovery/%	RSD/%		Recovery/%	RSD/%
Triamcinolone	65.8	3.8		87.3	5.7		78.9	6.5
Aldosterone	87.0	1.3		76.5	3.2		86.7	8.4
Prednisolone	84.2	2.6		74.4	1.9		92.8	3.5
Cortisol	70.5	0.7		75.5	6.0		80.0	2.2
Prednisone	94.3	1.8		86.6	1.3		94.5	6.1
Cortisone	72.1	3.5		68.0	0.4		72.6	4.0
Methylprednisolone	76.8	4.3		75.7	1.9		83.8	9.7
Betamethasone	73.3	4.4		81.4	2.1		73.6	2.9
Dexamethasone	74.0	5.4		78.0	2.7		88.3	12.6
Corticosterone	70.3	4.8		72.3	1.4		73.5	9.4
Flumethasone	77.6	3.3		89.0	0.7		92.5	5.4
Beclomethasone	66.0	6.3		80.8	3.6		78.7	7.1
Flunisolide	74.3	5.5		83.8	2.3		80.8	3.1
Triamcinolone acetonide	72.2	3.5		76.3	7.1		85.8	3.4
Fluocinolone acetonide	74.3	9.4		83.4	6.0		75.5	1.7
Fluorometholone	64.9	5.2		85.8	2.3		90.7	3.1
Fludrocortisone acetate	76.5	3.8		80.5	0.9		86.7	5.6
Deflazacort	71.3	2.2		75.8	3.4		125.1	8.6
Budesonide	70.4	3.4		75.1	7.8		71.7	9.5
Deoxycorticosterone acetate	79.2	6.3		69.5	1.5		86.1	11.8
Amcinonide	78.3	4.1		81.0	7.9		79.9	6.0
Clobetasol propionate	88.1	5.8		68.2	8.1		75.2	3.2
Fluticasone propionate	68.2	4.9		70.7	2.7		68.5	11.0
Clobetasone butyrate	79.2	5.4		85.9	4.4		96.7	5.1

### 2.7 实际样品分析

应用所建立的方法分析了3份取自珠江三角洲河流的沉积物样品,24种CSs测定结果见表4。样品中共有11种目标物(曲安西龙、倍他米松、地塞米松、曲安奈德、醋酸氟轻松、氟米龙、氟尼缩松、醋酸脱氧皮质酮、丙酸氯倍他索、丙酸氟替卡松和丁酸氯倍他松)被检出,含量范围为1.25~29.38 μg/kg。

**表 4:** 珠江三角洲河流沉积物样品中的皮质类固醇激素含量

Compound	Sample 1	Sample 2	Sample 3
Triamcinolone	3.78	<LOQ	ND
Aldosterone	ND	ND	ND
Prednisolone	ND	ND	ND
Cortisol	ND	ND	ND
Prednisone	ND	ND	ND
Cortisone	ND	ND	ND
Methylprednisolone	ND	ND	ND
Betamethasone	4.21	ND	<LOQ
Dexamethasone	9.63	ND	<LOQ
Corticosterone	ND	ND	<LOQ
Flumethasone	ND	ND	ND
Beclomethasone	ND	ND	ND
Triamcinolone acetonide	2.19	ND	ND
Fluocinolone acetonide	2.80	ND	ND
Fludrocortisone acetate	ND	ND	ND
Deflazacort	<LOQ	<LOQ	<LOQ
Fluorometholone	4.32	ND	ND
Budesonide	ND	ND	ND
Flunisolide	8.62	29.38	ND
Deoxycorticosterone acetate	7.51	22.35	1.28
Amcinonide	<LOQ	22.37	1.42
Clobetasol propionate	1.25	2.71	ND
Fluticasone propionate	2.03	1.85	ND
Clobetasone butyrate	ND	6.52	<LOQ

ND: not detected; <LOQ: less than limits of quantification.

## 3 结论

本研究建立了一种基于超声波提取-固相萃取技术对沉积物样进行前处理,应用UPLC-MS/MS同时检测沉积物中24种CSs的分析方法。该方法具有灵敏、准确和重现性好等特点,满足对沉积物中多种天然和合成CSs的痕量监测要求,可广泛应用到该类污染物环境行为和生态风险的研究中。
